# Association between Obstructive Sleep Apnea and Community-Acquired Pneumonia

**DOI:** 10.1371/journal.pone.0152749

**Published:** 2016-04-06

**Authors:** Eusebi Chiner, Mónica Llombart, Joan Valls, Esther Pastor, José N. Sancho-Chust, Ada Luz Andreu, Manuel Sánchez-de-la-Torre, Ferran Barbé

**Affiliations:** 1 Respiratory Department, Hospital Universitari Sant Joan d’Alacant, Alacant, Spain; 2 Biostatistics and Epidemiology Unit, IRB Lleida, Catalonia, Spain; 3 Respiratory Department, Hospital Universitari Arnau de Vilanova and Santa Maria, Universitat de Lleida, IRB Lleida, Catalonia, Spain; 4 Centro de Investigación Biomédica en Red de Enfermedades Respiratorias (CIBERES), Madrid, Spain; Charité—Universitätsmedizin Berlin, GERMANY

## Abstract

**Background:**

We hypothesized that obstructive sleep apnea (OSA) can predispose individuals to lower airway infections and community-acquired pneumonia (CAP) due to upper airway microaspiration. This study evaluated the association between OSA and CAP.

**Methods:**

We performed a case-control study that included 82 patients with CAP and 41 patients with other infections (control group). The controls were matched according to age, sex and body mass index (BMI). A respiratory polygraph (RP) was performed upon admission for patients in both groups. The severity of pneumonia was assessed according to the Pneumonia Severity Index (PSI). The associations between CAP and the Epworth Sleepiness Scale (ESS), OSA, OSA severity and other sleep-related variables were evaluated using logistic regression models. The associations between OSA, OSA severity with CAP severity were evaluated with linear regression models and non-parametric tests.

**Findings:**

No significant differences were found between CAP and control patients regarding anthropometric variables, toxic habits and risk factors for CAP. Patients with OSA, defined as individuals with an Apnea-Hypopnea Index (AHI) ≥10, showed an increased risk of CAP (OR = 2·86, 95%CI 1·29–6·44, p = 0·01). Patients with severe OSA (AHI≥30) also had a higher risk of CAP (OR = 3·18, 95%CI 1·11–11·56, p = 0·047). In addition, OSA severity, defined according to the AHI quartile, was also significantly associated with CAP (p = 0·007). Furthermore, OSA was significantly associated with CAP severity (p = 0·0002), and OSA severity was also associated with CAP severity (p = 0·0006).

**Conclusions:**

OSA and OSA severity are associated with CAP when compared to patients admitted to the hospital for non-respiratory infections. In addition, OSA and OSA severity are associated with CAP severity. These results support the potential role of OSA in the pathogenesis of CAP and could have clinical implications. This link between OSA and infection risk should be explored to investigate the relationships among gastroesophageal reflux, silent aspiration, laryngeal sensory dysfunction and CAP.

**Trial Registration:**

ClinicalTrials.gov NCT01071421

## Introduction

Obstructive sleep apnea (OSA) is a highly prevalent disorder affecting between 10–50% of middle-aged men [1]. OSA is characterized by recurrent upper airway obstruction associated with cyclic changes in oxyhemoglobin saturation, intermittent arousals from sleep and alterations in intrathoracic pressure. These alterations induce oxidative stress, sympathetic activation, and metabolic dysregulation [2]. OSA is also related to the increased incidence of hypertension, cardiovascular events [3,4] and cancer [5].

A small amount of silent aspiration during the night is normal in healthy individuals [6]. However, OSA can predispose an individual to increased aspiration [7]. OSA is characterized by alterations in the intrathoracic pressure and by a weak or absent cough reflex, mainly during rapid eye movement (REM) sleep [8,9] These alterations may be due to the action of cortical inhibitory nerves to the brainstem cough center and an increased threshold of peripheral nerves at night. Gastroesophageal reflux (GER) is frequently associated with OSA [10,11], and previous studies have suggested a possible association between OSA and respiratory disorders, such as cough, pneumonia, asthma or laryngitis [12,13]. Furthermore, treatment of GER produces small but significant reductions in the apnea hypopnea index (AHI) [14,15], and studies have demonstrated that many patients who do not respond to anti-reflux therapies experience sleep-disordered breathing [10].

Four recent studies, one involving children <5 years of age [16] and three national surveys among adults [17–19], have suggested that OSA could increase the incidence of pneumonia. To address this question, we designed a case-control study to compare the prevalence of OSA among patients with community-acquired pneumonia (CAP) with respect to patients with other non-respiratory infections. To our knowledge, this report is the first evaluation of the risk of CAP in OSA patients.

## Methods

### Study design

A case-control study was performed at the University Hospital of San Juan de Alicante, Spain, from 2012 to 2014. Cases were defined as patients admitted to the hospital with a diagnosis of CAP. Controls were defined as patients admitted to the Departments of Internal Medicine, Infectious Diseases or Traumatology for other infectious diseases not related to the respiratory tract. Patients with infections were included in both groups (cases and controls) to ensure that these groups were as similar as possible. The presence of infection, regardless of an infection’s origin, implies an increase in systemic inflammatory activity. This inflammation could be related to the presence of OSA. Patients with suspected bronchogenic carcinoma, human immunodeficiency virus (HIV) infection or acquired immunodeficiency syndrome (AIDS), severe acute cardiorespiratory comorbidity, nosocomial pneumonia, neurological deficits, other sleep disorders (dyssomnias or parasomnias), patients previously diagnosed of OSA, CPAP treatment, use of hypnotics or sedatives and those without informed consent were excluded. Clinical interviews were used to exclude patients with other sleep disorders. In addition, for controls, patients with infections of the ear, nose and throat (ENT), such as rhinosinusitis, tonsillitis or otitis, were also excluded. Cases and controls were matched at a 2:1 ratio according to age, sex and body mass index (BMI); in particular, pairs of cases with similarities in age, sex and BMI (minimizing the differences within pairs) were first established, and a matched control was then selected.

### Primary association: OSA as a risk factor for pneumonia

OSA and OSA severity were assessed as risk factors for the presence of CAP according to different thresholds in the AHI scale (AHI ≥10 vs. AHI <10, AHI ≥30 vs. AHI <30 and AHI quartiles). The associations between the Epworth Sleepiness Scale (ESS), other sleep-related variables with CAP were also assessed.

### Secondary association: OSA and pneumonia severity

The associations between OSA, and OSA severity with pneumonia severity were assessed according to different thresholds in the AHI scale (AHI ≥10 vs. AHI <10, AHI ≥30 vs. AHI <30 and AHI quartiles). The severity of pneumonia was defined by the pneumonia severity index (PSI) [20]. This association was specifically assessed for CAP cases.

### Protocol and procedures

Patients with CAP were evaluated within the first 48 hours after hospitalization. Specific sleep questionnaires were used to evaluate symptoms related to OSA. Daytime sleepiness was evaluated with the ESS [21]. Demographic aspects, toxic habits (smoking, ex-smoking, alcohol habit), and comorbidities (COPD, diabetes, oral or inhaled steroids during the last 6 months, previous pneumonia, asthma, heart failure, kidney failure, hospitalization during the previous 3 months, cerebrovascular disease, lung neoplasms, extrapulmonary neoplasms and bronchiectasis) were assessed. Physical examinations were conducted, including weight, height and the Mallampati scale.

CAP was defined as the presence of an infiltrate (observed via a chest X-ray) and an acute illness associated with one or more of the following signs and symptoms: new cough with or without sputum production, pleuritic chest pain, dyspnea, fever, hypothermia, altered breath sounds on auscultation or leukocytosis [22]. The CAP admission criteria were based on the Spanish guidelines [23]. CAP assessment included a blood analysis, arterial blood gases and quantitative cultures from sputum or bronchial-protected samples obtained by fiberoptic bronchoscopy. The etiological diagnosis was investigated in all cases.

A validated recording device system was used to measure respiratory disturbance during sleep in patients and controls (Stardust®, Respironics, Murrisville, CA, USA) [24]. Scoring was performed manually by researchers blinded to the allocation of patients into groups. The AHI scale was defined as the number of apneas + hypopneas divided by the number of hours in bed or recording time. The minimum recording time was 6 hours, and the minimum time for valid analysis was 240 minutes. Oximetric parameters were also obtained from oximetry tracings, including the baseline oxygen saturation (SpO_2_ basal), medium oxygen saturation (SpO_2_ med), minimum oxygen saturation (SpO_2_ min) and percentages below saturation between different intervals as a percentage of total time with O_2_ saturation <90% (Tsat_90_). An AHI value ≥10 was considered to be diagnostic for OSA, and AHI≥30 was considered to represent severe OSA. The OSA threshold of AHI ≥10/h was chosen based on a cutoff with good sensitivity and specificity in ROC curves. In particular, S = 74% and E = 100% for an AHI of 15/h in polysomnography; this value was based on previous work and corresponded to significant OSA [25].

A respiratory polygraph (RP) was performed on supplemental oxygen as needed (38 cases, 47%) based on each patient’s medical condition. In patients with CAP, the sleep study was repeated at home 1 month later.

### Ethical aspects

The study was approved by the Ethics and Clinical Trials Committee of the San Juan University Hospital, Alicante, Spain (05/104-1) in 2010, and written informed consent was obtained from all patients before inclusion. The study began in 2012 and was completed in 2014. This study is registered at the clinical trials registry (www.clinicaltrials.gov) with the number NCT01071421. The authors confirm that all ongoing and related trials for this intervention are registered.

### Statistical analyses

Means (and standard errors) and absolute frequencies (and percentages) were used to describe the quantitative and qualitative variables, respectively. First, we compared the baseline variables between CAP cases and controls using the Mann-Whitney or Fisher test. The intra-class correlation coefficient (ICC) was computed to assess the concordance between sleep measurements during hospitalization and at home, and the 95% confidence intervals (CIs) were obtained. Second, the association between the ESS, OSA, OSA severity (AHI and the AHI quartiles considered on quantitative scales), and other sleep-related variables with the risk of CAP was assessed. For this purpose, logistic regression models were used to estimate the differences in CAP risk and calculate the odds ratios (OR), corresponding 95% CIs and p-values. A trend test was also performed to specifically evaluate the association of OSA severity (according to AHI quartiles) with CAP risk based on the logistic regression model. Finally, a specific analysis was performed to assess the association between OSA, OSA severity and CAP severity (with PSI as the quantitative scale). Thus, the Mann-Whitney and Kruskal-Wallis tests were used to compare the PSI levels among the different groups based on AHI thresholds (≥10, ≥30 and quartiles). A linear regression model was used to evaluate the increased severity in PSI levels according to the AHI quartiles. In addition, a Pearson correlation index was calculated to evaluate the linear relationship between AHI and PSI, and a Spearman test was used to assess significance. All analyses were performed using the R statistical package (version 3·2·1, 2015, http://www.R-project.org/) and the threshold for significance was set at 5% (α = 0·05). The results described in the text are mainly supported by tables indicating the results obtained using the statistical methods described above. Certain figures that emphasize significant effects and contribute to visualizing the sizes of these effects are also provided.

### Sample size and statistical power

We determined that the inclusion of 82 CAP cases and 41 controls would be required to achieve 80% statistical power (β = 0·2) and detect a significant difference (OR at least 2·5 units) to assess the association between cases and controls with pneumonia risk (assuming a prevalence of OSA in the control group of 40%). Using for this purpose a one-sided Z test based on two proportions, and setting the significance level at 5% (α = 0·05).

## Results

### Patients

**Fig 1:**
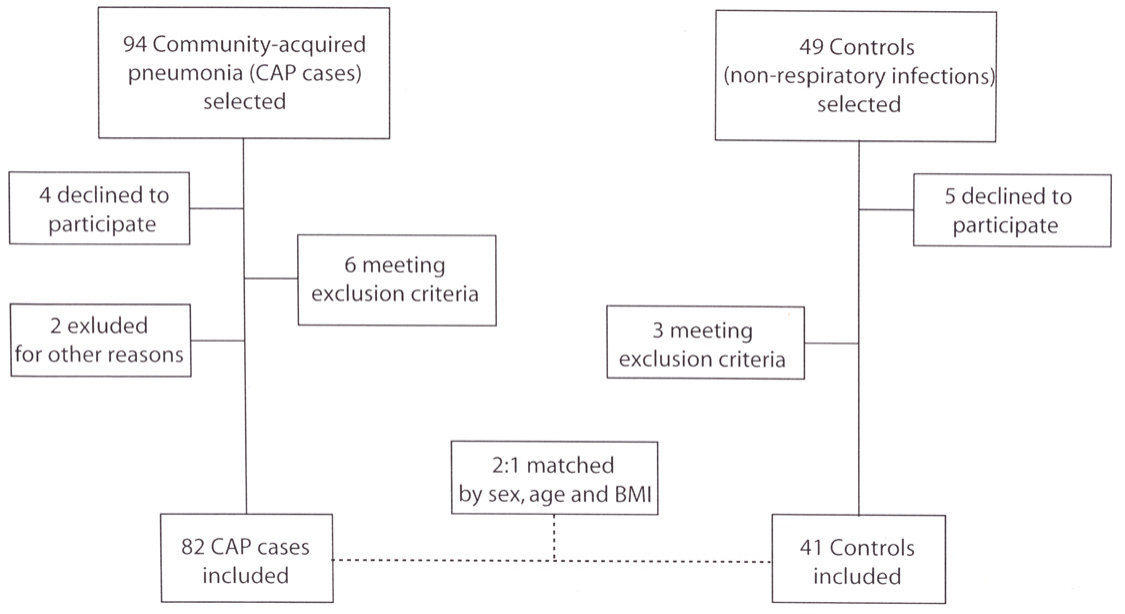
Study flowchart depicting recruitment.

Fig 1 shows the flowchart for the study participants according to the STROBE (Strengthening the Reporting of Observational Studies in Epidemiology) recommendations for case-control epidemiological studies.

In total, 82 CAP cases were included in the study. Therefore, 41 control patients who were admitted to the hospital for various non-respiratory infections were selected. Cases and controls (in a 2:1 ratio) were matched according to age, sex and BMI. The severity of pneumonia, invasive diagnostic techniques used and bacterial etiology for the patients in the CAP group are described in Table 1.

**Table 1:** Baseline clinical characteristics, invasive diagnostic techniques and bacterial etiology in the CAP group.

	CAP cases (n = 82)
PSI score, mean	75.94 (30.71)
Class I-III	57 (69.51%)
Class IV	21 (25.61%)
Class V	4 (4.88%)
Invasive diagnostic techniques	
Fiberoptic bronchoscopy samples	34 (41.46%)
Thoracocentesis	5 (6.10%)
Other	43 (52.44%)
Etiological diagnosis^a^	39 (47.56%)
Streptococcus pneumoniae	15 (38.46%)
Mycoplasma pneumoniae	7 (17.95%)
Pseudomonas aeruginosa	4 (10.26%)
Legionella pneumophila	3 (7.69%)
Staphylococcus aureus	3 (7.69%)
Streptococcus viridans	1 (2.56%)
Coxiella burnetti	1 (2.56%)
Escherichia coli	1 (2.56%)
Chlamydia pneumoniae	1 (2.56%)
Streptococcus oralis	1 (2.56%)
Streptococcus salivarius	1 (2.56%)
Streptococcus sanguis	1 (2.56%)
Mean Hospital stay, days	7.67± 3.7
Admission in ICU	3 (3.66%)

Data are presented as means (standard deviations) or frequencies (percentages).

^a^ Six patients presented with more than one diagnosis. The percentage for each diagnosis was calculated with respect to the total number of diagnoses (n = 39).

The different infection diagnoses for the patients in the control group are described in Table 2.

**Table 2:** Diagnoses in the control group.

	Controls (n = 41)
Diagnosis	
Urinary tract infection	7 (17.07%)
Acute pyelonephritis	7 (17.07%)
Acute gastroenteritis	5 (12.20%)
Cellulitis of the extremities	5 (12.20%)
Fever of unknown origin	4 (9.76%)
Urinary sepsis	3 (7%)
Prostatitis	2 (4.88%)
Botulism	2 (4.88%)
Lymph node tuberculosis	1 (2.44%)
Cholecystitis	1 (2.44%)
Liver abscess	1 (2.44%)
Biliary sepsis	1 (2.44%)
Prosthetic knee infection	1 (2.44%)
Osteomyelitis	1 (2.44%)

Frequencies and percentages are shown for each infection.

### Concordance between sleep respiratory measurements obtained during hospitalization and at home

Acceptable correlations between sleep respiratory measurements acquired during hospitalization and at home were observed for AHI, AI (apnea index), mean SaO_2_ and minimum SaO_2_ (ICCs of 0.41, 0.45, 0.39 and 0.36, respectively) (Table 3). The HI (hypopnea index), oxygen desaturation index (ODI) and time spent with SaO_2_< 90% (TC90) were slightly higher at home, but this difference was not significant.

**Table 3:** Concordance analysis for the sleep respiratory measurements obtained during hospitalization and at home for CAP patients.

	CAP cases (n = 82)		Intra-class correlation coefficient
	During admission (n = 82)	At home (n = 56)*	
**Apnea-hypopnea index (h**^**-1**^**)**	19.07 (13.68)	23.94 (17.46)	0.41 (0.16,0.59)
**Apnea index**	14.06 (12.59)	17.87 (15.17)	0.45 (0.21,0.62)
**Hypopnea index**	4.88 (5.71)	6.02 (7.28)	0.05 (-0.28,0.31)
**ODI**	12.88 (11.96)	16.69 (14.48)	0.28 (-0.01,0.49)
**Mean SaO**_**2**_**(%)**	93.81 (3.16)	93.7 (2.92)	0.39 (0.13,0.58)
**Minimum SaO**_**2**_**(%)**	81.15 (9.4)	80.18 (9.64)	0.36 (0.08,0.55)
**Tsat**_**90**_	10.39 (21.07)	8.19 (17.41)	0.17 (-0.14,0.41)

Means (and standard deviations) for each measurement recorded during admission and at home are shown. Intra-class correlation coefficients and 95% CIs are shown.

* The home study could not be performed in 26 patients: 14 refused, 5 cited social problems, 3 were invalid, 2 moved to a different house and 2 died in the intensive care unit.

### Homogeneity of cases and controls

No significant differences were found between CAP and control patients regarding sex, age and BMI (Table 4). No significant differences were found between CAP and control patients regarding other baseline anthropometric variables, toxic habits and risk factors for CAP. Therefore, both groups were clinically homogeneous (controlling for potentially confounding variables and risk factors).

**Table 4:** Differences between patients with CAP and patients with other infections (controls): baseline anthropometric variables, toxic habits and risk factors for CAP.

	Total (n = 123)	Control (n = 41)	CAP (n = 82)	p-value
Age	61·27 (18·86)	58·61 (17·96)	62·60 (19·26)	0·22
Sex (Men)	56 (45·53%)	18 (43·9%)	38 (46·34%)	0·85
Body mass index	26·70 (4·20)	26·42 (4·73)	26·84 (3·94)	0·39
Neck circumference	39·84 (3·16)	39·68 (3·50)	39·91 (3·00)	0·95
Smoking				0·22
Non-smoker	60 (48·78%)	23 (56·1%)	37 (45·12%)	
Former-smoker	26 (21·14%)	5 (12·2%)	21 (25·61%)	
Smoker	37 (30·08%)	13 (31·71%)	24 (29·27%)	
Alcohol intake > 40 g/day	27 (21·95%)	8 (19·51%)	19 (23·17%)	0·82
COPD	21 (17·07%)	4 (9·76%)	17 (20·73%)	0·2
Asthma	11 (8·94%)	1 (2·44%)	10 (12·2%)	0·1
Bronchiectasis	7 (5·69%)	0 (0%)	7 (8·54%)	0·09
Previous pneumonia	12 (9·76%)	1 (2·44%)	11 (13·41%)	0·06
Chronic heart failure	8 (6·5%)	1 (2·44%)	7 (8·54%)	0·27
Diabetes mellitus	20 (16·26%)	7 (17·07%)	13 (15·85%)	1
Chronic renal failure	6 (4·88%)	0 (0%)	6 (7·32%)	0·18
Neoplasm	4 (3·25%)	3 (7·32%)	1 (1·22%)	0·11
Cerebrovascular diseases	5 (4·07%)	0 (0%)	5 (6·1%)	0·17
Steroid treatment	13 (10·57%)	1 (2·44%)	12 (14·63%)	0·06
Admission in 3 month previous	9 (7·32%)	3 (7·32%)	6 (7·32%)	1
Comorbidity*	90 (73.2%)	27 (65.9%)	63 (76.8%)	0.2

Data are presented as the means (and standard deviations) and absolute frequencies (and percentages) for quantitative and qualitative variables, respectively. P-values were obtained with Mann-Whitney U tests or Fisher’s exact tests to assess the differences between cases and controls.

*Patients with at least 1 comorbidity described in the table.

### Association of OSA and OSA severity with CAP

**Fig 2:**
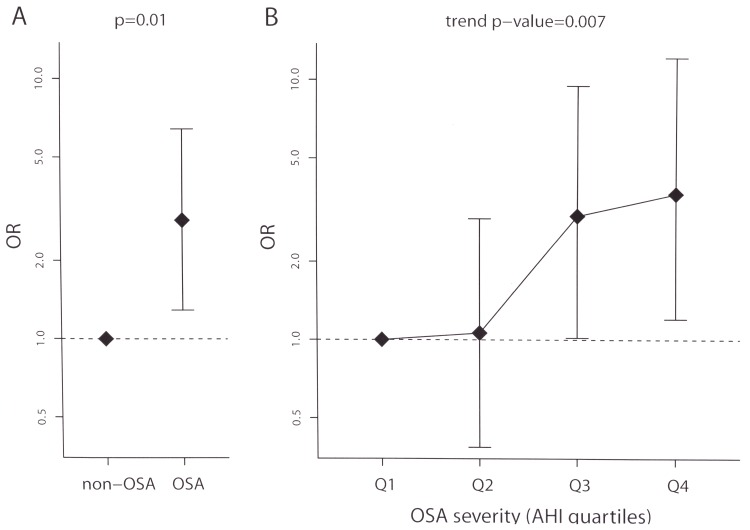
**Association of OSA (A) and OSA severity (B) with CAP.** ORs and corresponding 95% CIs (segments) comparing the risk of CAP in (A) patients with OSA (AHI≥10 vs. AHI<10) and (B) patients according to OSA severity (AHI quartiles) are shown. P-values from a logistic regression model and from a trend test are also shown.

No significant differences were found in the sleepiness levels measured with the Epworth questionnaire between groups (Table 5). Patients with OSA, defined as AHI≥10, had a significant 2·86-fold increase in the risk of CAP, p = 0·01 (Table 5 and Fig 2A). Patients with severe OSA, defined as AHI≥30, had a significant 3·18-fold increase in the risk of CAP (Table 5). In addition, AHI levels were significantly higher in CAP patients (Table 5). OSA severity, defined by the AHI quartile, was significantly associated with CAP (ORs 1·06, 3 and 3·65 for Q2, Q3 and Q4, respectively, using Q1 as the reference, p = 0·007, Table 5, Fig 2B). Furthermore, all indices of oxygen saturation were significantly worse in the CAP group (Table 5).

**Table 5:** Associations of ESS, OSA, OSA severity, and other sleep-respiratory variables with CAP.

	Total (n = 123)	Control (n = 41)	CAP (n = 82)	OR (95% CI)	p-value
**Epworth Sleepiness Scale**	6·39 (3·48)	6·80 (3·03)	6·20 (3·69)	0·951 (0·852;1·061)	0·37
**OSA**					
**AHI <10 (non-OSA)**	38 (30·89%)	19 (46·34%)	19 (23·17%)	1	
**AHI>10 (OSA)**	85 (69·11%)	22 (53·66%)	63 (76·83%)	2·864 (1·291;6·44)	0·01
**AHI<30**	98 (79·67%)	37 (90·24%)	61 (74·39%)	1	
**AHI >30 (severe OSA)**	25 (20·33%)	4 (9·76%)	21 (25·61%)	3·184 (1·108;11·556)	0·047
**OSA severity**					
**AHI, quantitative scale**	19·07 (13·68)	14·81 (11·41)	21·20 (14·27)	1·042 (1·009;1·08)	0·02
**AHI, quartiles**					
**Q1 (AHI 0–9·2)**	30 (24·39%)	14 (34·15%)	16 (19·51%)	1	
**Q2 (AHI 9·3–14·1)**	31 (25·2%)	14 (34·15%)	17 (20·73%)	1·063 (0·386;2·929)	0·91
**Q3 (AHI 14·2–27·5)**	31 (25·2%)	7 (17·07%)	24 (29·27%)	3 (1·018;9·498)	0·052
**Q4 (AHI 27·6 or higher)**	31 (25·2%)	6 (14·63%)	25 (30·49%)	3·646 (1·203;12·169)	0·03
				Trend p-value = 0·007	
**Other sleep-related variables:**					
**ODI**	12·88 (11·96)	9·56 (9·02)	14·54 (12·91)	1·043 (1·006;1·088)	0·03
**Mean SaO**_**2**_	93·81 (3·16)	94·73 (1·82)	93·35 (3·57)	0·852 (0·733;0·973)	0·03
**Minimum SaO**_**2**_	81·15 (9·40)	83·78 (7·66)	79·84 (9·94)	0·948 (0·899;0·992)	0·03
**TC90, %**	10·39 (21·07)	2·36 (6·51)	14·41 (24·47)	1·065 (1·022;1·144)	0·02

Two AHI-based thresholds (above 10 or 30) and AHI quartiles were used to assess the association of OSA severity with CAP. Data are presented as the means (and standard deviations) and absolute frequencies (and percentages) for quantitative and qualitative variables, respectively. Logistic regression models were used to assess the differences in CAP risk. The ORs, corresponding 95% CIs and p-values were calculated. P-values from a trend test to assess the association between OSA severity and CAP are also shown.

### Association of OSA and OSA severity with CAP severity

**Fig 3:**
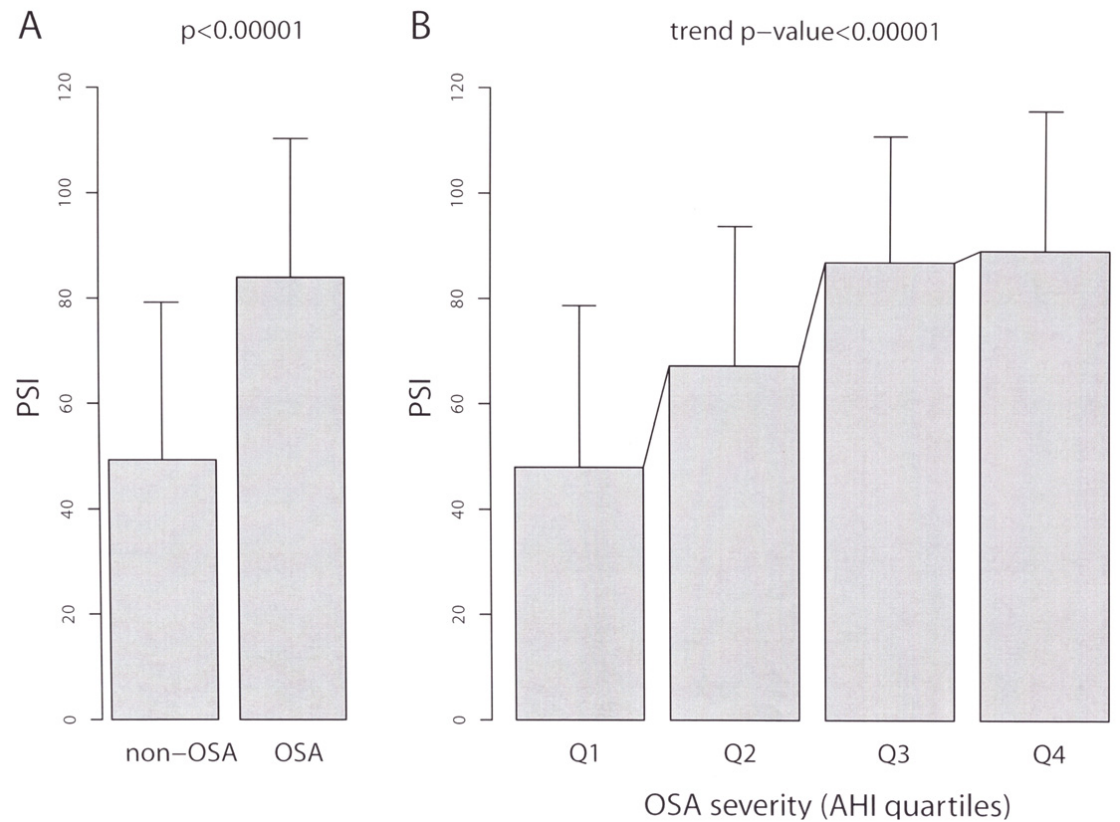
**Differences in CAP severity (PSI) according to an (A) AHI cutoff of 10 and (B) quartiles classification.** Bar height represents the mean PSI value, and the segments represent one standard deviation for each category considered.

Specific analysis of the 82 CAP patients revealed that OSA was significantly associated with CAP severity (PSI means 83·95 ± 26·30 and 49·37 ± 29·83 in patients with AHI≥10 and AHI<10, respectively, p = 0·0002, Table 6 and Fig 3A). In addition, OSA severity was clearly associated with pneumonia severity. Indeed, the AHI levels were significantly and positively correlated with the PSI levels (Pearson correlation r = 0·43, p<0·00001, and the mean PSI levels were increased according to the different OSA grades (PSI means 48·00 ± 30·66, 67·24 ± 25·56, 86·96 ± 23·86 and 89·16 ± 26·49, respectively in the AHI quartiles, p<0·00001, Table 6 and Fig 3B).

**Table 6:** Association of OSA and OSA severity with CAP severity.

	PSI (n = 82)	p-value
**OSA**		
**AHI <10 (non-OSA)**	49·37 (29·84)	
**AHI >10 (OSA)**	83·95 (26·30)	0·00002^a^
**AHI<30**	70·95 (30·03)	
**AHI >30 (severe OSA)**	90·43 (28·58)	0·02^a^
**OSA severity**		
**AHI, quantitative scale**	0·43 (0·19)*	<0·00001^b^
**AHI, quartiles**		
**Q1 (AHI 0–9·2)**	48·00 (30·66)	
**Q2 (AHI 9·3–14·1)**	67·24 (26·56)	
**Q3 (AHI 14·2–27·5)**	86·96 (23·86)	
**Q4 (AHI 27·6 or higher)**	89·16 (26·49)	0·00006^c^
	Trend p-value<0·00001^d^	

The PSI was used to assess CAP severity. Two AHI-based thresholds (above 10 or 30) were used to assess the association between OSA and CAP severity. AHI (quantitative scale and in quartiles) was used to assess the association between OSA severity and CAP severity. Data are presented as the means (and standard deviations).

* Pearson correlation coefficients (and R-squared) are shown for AHI (quantitative scale). P-values to assess the association with CAP severity were computed using Mann-Whitney U tests (a), Spearman’s correlation test (b), a Kruskal-Wallis test (c) and a linear regression model (using AHI quartiles as an ordinal variable with integer values) (d).

## Discussion

The presence of OSA almost triples the risk of CAP, and this increased risk is related to the severity of OSA. Additionally, CAP severity is related to OSA severity. These findings suggest that OSA could be related to the pathogenesis of CAP.

There is a paucity of studies on the relationship of OSA and lung infections, although recent studies have addressed this issue. Goldbart et al. showed that a previous diagnosis of OSA was more common in children <5 years with pneumonia compared to children without pneumonia [16]. Su et al. observed a 1·2-fold increase in the incidence of pneumonia in adult patients with OSA (from the Taiwan National Health Insurance Research Database) [17]. Lindenauer et al. carried out a retrospective cohort study of patients with pneumonia at 347 US hospitals, including a total of 250,907 patients, showing that OSA is associated with higher rates of mechanical ventilation, increased risk of clinical deterioration, higher resource use, and a modestly lower risk of inpatient mortality [18]. Jean et al. carried out a retrospective study in 74,032 patients with pneumonia and invasive mechanical ventilation, showing that OSA patients had a significantly shorter hospital stay and a reduced risk of mortality. However [19], the lack of individual data and the potential confounding factors not assessed in these studies limits the interpretation of their results. Furthermore, these retrospective studies only analyze the outcomes in patients with a prior diagnosis of OSA, but unlike our study did not assess which patients with pneumonia may have OSA undiagnosed. Our case control study overcomes this limitation and reveals a clear association between OSA and CAP.

OSA is associated with upper airway inflammation and laryngeal inflammation and is correlated with laryngeal sensory dysfunction and laryngeal adductor reflection attenuation [13]. Additionally, OSA is related to a weak or absent cough reflex, mainly during REM sleep [8,9], stasis of the bolus after swallowing [26] and gastroesophageal reflux [10,11]. Our hypothesis is that OSA may be the link between abnormal AHI and silent microaspiration that causes CAP. Together, these alterations may induce a change in the oropharyngeal microflora, producing silent aspiration and leading to inflammatory phenomena of the lower airway. This inflammation may trigger the systemic inflammatory factors and hypoxia-re-oxygenation that is typically found in patients with altered AHI [27]. Finally, this succession and interrelation of events can cause pneumonia, as we have demonstrated in our work. Aspiration during sleep, as measured by radioactive 99mTc tracers, has been demonstrated in healthy [28] and OSA individuals [29]. However, the large quantity aspirated by OSA individuals likely contains bacterial organisms in physiologically significant quantities. Moreover, the microbiota composition and diversity of the intestines are altered as a result of intermittent hypoxia mimicking OSA, suggesting that physiological interplays between host and gut microbiota could be deregulated in patients with OSA [30], and this dysregulation may also contribute to the pathogenesis of pneumonia in OSA patients. This link between OSA and infection risk should be explored to investigate the relationships among gastroesophageal reflux, silent aspiration, laryngeal sensory dysfunction and CAP.

The lack of somnolence in patients with OSA and the diagnostic symptoms suggest that the only way to diagnose OSA is by performing a sleep study. Our results confirm this suggestion. Additionally, a previous history of CAP and the use of inhaled or oral steroids during the previous 6 months are related to a higher prevalence of current CAP.

Our study had some limitations. First, this study was conducted exclusively with patients admitted to our hospital, and the results cannot be extrapolated to outpatients with CAP. Additionally, both populations likely have an increased risk of comorbidities, which could have caused the high prevalence of abnormal AHI in our population. However, we observed that the prevalence of abnormal AHI in the control group was similar to this prevalence in the general population [1]. Second, RP was performed during the admission period with supplementary O_2_ in some patients when medically necessary. However, O_2_ administration only mildly interferes with AHI calculations [31]. In addition, when RP was performed at home during the clinically stable period, AHI was not significantly different.

In conclusion, OSA is associated with CAP requiring hospital admission. In addition, OSA and OSA severity are associated with CAP severity. These results support the potential role of OSA in the pathogenesis of CAP, and this role should be explored in large-scale, multicenter studies.

## Supporting Information

S1 TREND Checklist (PDF)

S1 File Full access to the database of this study. (SAV)
